# Samovar: Single-Sample Mosaic Single-Nucleotide Variant Calling with Linked Reads

**DOI:** 10.1016/j.isci.2019.05.037

**Published:** 2019-05-29

**Authors:** Charlotte A. Darby, James R. Fitch, Patrick J. Brennan, Benjamin J. Kelly, Natalie Bir, Vincent Magrini, Jeffrey Leonard, Catherine E. Cottrell, Julie M. Gastier-Foster, Richard K. Wilson, Elaine R. Mardis, Peter White, Ben Langmead, Michael C. Schatz

**Affiliations:** 1 Department of Computer Science, Johns Hopkins University, Baltimore, MD, USA; 2 The Institute for Genomic Medicine, Nationwide Children's Hospital, Columbus, OH, USA; 3 Department of Pediatrics, The Ohio State University College of Medicine, Columbus, OH, USA; 4 Department of Neurosurgery, Nationwide Children's Hospital, Columbus, OH, USA; 5 Department of Biology, Johns Hopkins University, Baltimore, MD, USA; 6 Cold Spring Harbor Laboratory, Cold Spring Harbor, NY, USA

**Keywords:** Biological Sciences, Genomics, Bioinformatics

## Abstract

Linked-read sequencing enables greatly improves haplotype assembly over standard paired-end analysis. The detection of mosaic single-nucleotide variants benefits from haplotype assembly when the model is informed by the mapping between constituent reads and linked reads. Samovar evaluates haplotype-discordant reads identified through linked-read sequencing, thus enabling phasing and mosaic variant detection across the entire genome. Samovar trains a random forest model to score candidate sites using a dataset that considers read quality, phasing, and linked-read characteristics. Samovar calls mosaic single-nucleotide variants (SNVs) within a single sample with accuracy comparable with what previously required trios or matched tumor/normal pairs and outperforms single-sample mosaic variant callers at minor allele frequency 5%–50% with at least 30X coverage. Samovar finds somatic variants in both tumor and normal whole-genome sequencing from 13 pediatric cancer cases that can be corroborated with high recall with whole exome sequencing. Samovar is available open-source at https://github.com/cdarby/samovar under the MIT license.

## Highlights

• Samovar uses haplotype-specific features from linked reads to call mosaic variants
• Samovar quickly evaluates candidates with a random forest over 33 features
• Only one sample is needed, with accuracy comparable with paired samples or trios
• Samovar finds somatic variants in cancer driving genes in 13 pediatric cancer cases

## Graphical Abstract

**Figure:**
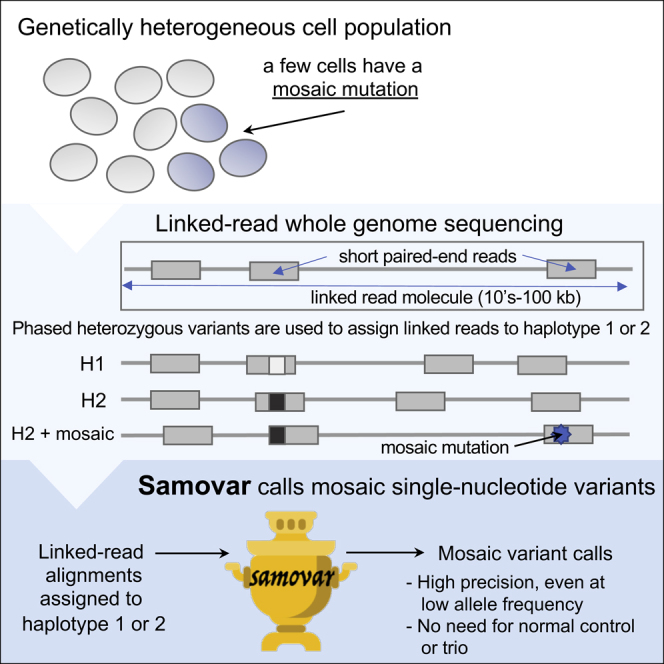

## Introduction

Genomic mosaicism results from postzygotic *de novo* mutations, ranging from single-nucleotide changes to larger structural variants and whole chromosome aneuploidy. Mosaic mutations are present in some of the cells belonging to the offspring but in none of either parents' cells (Biesecker and Spinner, 2013, Cohen et al., 2015). The distribution and prevalence of cells with a mosaic mutation depend on a combination of the developmental cell lineage, stage at which the mutation occurred, selection for or against cells with the mutation (Youssoufian and Pyeritz, 2002), and cell migration (Freed et al., 2014). Somatic mosaicism refers to genetic heterogeneity among non-germ cells, which accrue in normally dividing cells throughout the human lifetime (Gajecka, 2016, Laurie et al., 2012, Kennedy et al., 2012) corroborated by monozygotic twin studies (Ouwens et al., 2018). Mosaicism also plays an important role in many genetic diseases. Pathologically, cancer is characterized by an overall increased mutational load in tumor cells as well as a high level of intra-tumor genetic heterogeneity (Vogelstein et al., 2013, Watson et al., 2013). Mosaicism has also been implicated in autism (Freed and Pevsner, 2016) and is being explored in connection to other neurological disease (Poduri et al., 2013, McConnell et al., 2017, D’Gama and Walsh, 2018). Causal mosaic mutations have also been found for Sturge-Weber syndrome (Shirley et al., 2013), McCune-Albright syndrome (Weinstein et al., 1991), and Proteus syndrome (Lindhurst et al., 2011), among others.

Mosaic variants can be detected by whole-genome or targeted sequencing of affected tissue. Samovar operates on linked reads, which are sets of sequencing reads deriving from a longer fragment such as those from the 10X Genomics Chromium instrument (Pleasanton, CA, USA). Although the individual (“constituent”) reads are typical short Illumina reads, the longer fragments can be tens or hundreds of kilobases long. The mapping from constituent reads to fragments of origin is established by molecular barcodes added in the Chromium library preparation step. The average sequencing coverage per long fragment is usually low: around 0.1-fold (Zheng et al., 2016, Marks et al., 2019). Since constituent reads can be paired-end, we use the term “long fragment” for the longer fragment from which a linked read is derived and “short fragment” for fragments from which paired-end reads are derived.

**Figure 1:**
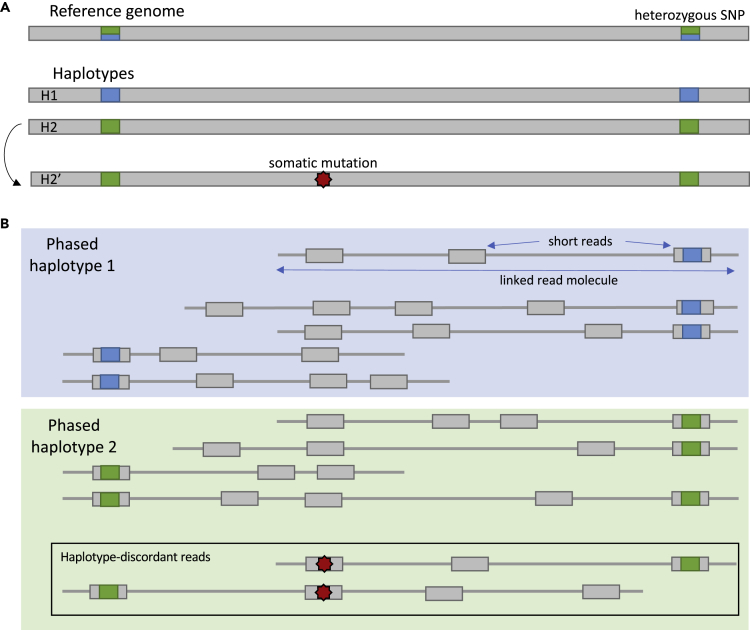
Schematic Representation of Somatic Mutations within a Phased Sample (A) A mosaic mutation occurs on haplotype H2. (B) Therefore, in linked-read sequencing, where short reads can be phased when linked reads overlap phased heterozygous variants, mosaic mutations manifest on reads from only one haplotype, here H2. Adapted from Figure 3 of Dou et al., 2018.

The properties of linked reads enable many potential improvements in variant detection and related analyses (Sedlazeck et al., 2018). For example, a constituent read that would align repetitively by itself might align uniquely when alignments of other reads from the same long fragment are accounted for (Bishara et al., 2015, Shajii et al., 2018). Linked-read-based algorithms have been developed for *de novo* assembly (Kuleshov et al., 2016, Weisenfeld et al., 2017, Mostovoy et al., 2016), *de novo* mutation calling (Zhou et al., 2018), assembly error correction (Jackman et al., 2018), and structural variant calling (Elyanow et al., 2018, Xia et al., 2018, Spies et al., 2017, Eslami Rasekh et al., 2017, Fang et al., 2018). Also, linked reads enable more accurate and contiguous assembly of haplotypes (Zheng et al., 2016, Marks et al., 2019, Edge et al., 2017) since constituent reads can be phased even when only some overlap heterozygous variants (Figure 1B).

Although downstream tools benefit automatically from some linked-read properties, e.g., improved alignment accuracy, other benefits require specialized methods to exploit. In particular, the detection of a somatic mosaic single-nucleotide variant (SNV) can benefit from haplotype assembly when the variant detection model is informed by the mapping between constituent reads and linked reads. As an example, in a diploid sample with haplotypes H1 and H2, suppose a mosaic mutation occurs on haplotype H2 yielding a collection of reads (labeled H2′) that have the mosaic allele but otherwise match H2 (Figure 1A). The mosaic mutation will likely be tolerated by the haplotype assembler, and the reads will still be assigned to H2 (Figure 1B). The fact that all the mosaic-carrying reads fall on the same haplotype is a hallmark of post-zygotic mosaicism (Freed and Pevsner, 2016) and contrasts with sequencing error, which would tend to distribute the “mosaic” alleles evenly across haplotypes (Usuyama et al., 2014). Reads with the mosaic allele are called haplotype-discordant reads, and these are the most reliable kind of evidence we can gather in support of mosaic variants.

The mosaic variant caller's task is to distinguish the signature of a mosaic variant from that of a germline variant after it has been affected by sequencing errors, alignment errors, copy-number changes, and other confounders. Most methods employ statistical tests on the sequencing reads aligned to a particular site, comparing allele frequency between “tumor” and “normal” (or between the observed and expected value for a germline variant). HapMuC (Usuyama et al., 2014) uses haplotype phasing of nearby heterozygous germline variants in conjunction with a tumor-normal pair to call somatic variants, but local phasing is limited by read length of paired-end short reads. In single-cell linked-read data, LiRA (Bohrson et al., 2019) leverages heterozygous germline variants and the additional locality information of linked reads to call mosaic SNVs. See Dou et al., 2018 for a review of methods to detect such mutations in scenarios other than cancer and Wang et al., 2013 for a comparison of several tools in the cancer context. Samovar is unique in that it is the first to evaluate haplotype-discordant reads identified through linked-read sequencing, thus enabling phasing and mosaic variant detection across essentially the entire genome. It also evaluates the statistical characteristics of the haplotypes, depth of coverage, and potential confounders such as alignment errors to robustly identify mosaic variants from a single sample.

## Results

### Samovar Pipeline

We present Samovar, a single sample mosaic SNV caller designed for 10X Genomics linked-read whole-genome sequencing (WGS) data. Samovar takes as input phased variants in VCF format and linked-read alignments in BAM format. These are both output by 10X Genomics' Long Ranger pipeline, which preprocesses reads, aligns linked reads, calls variants, and assembles haplotypes.

**Figure 2:**
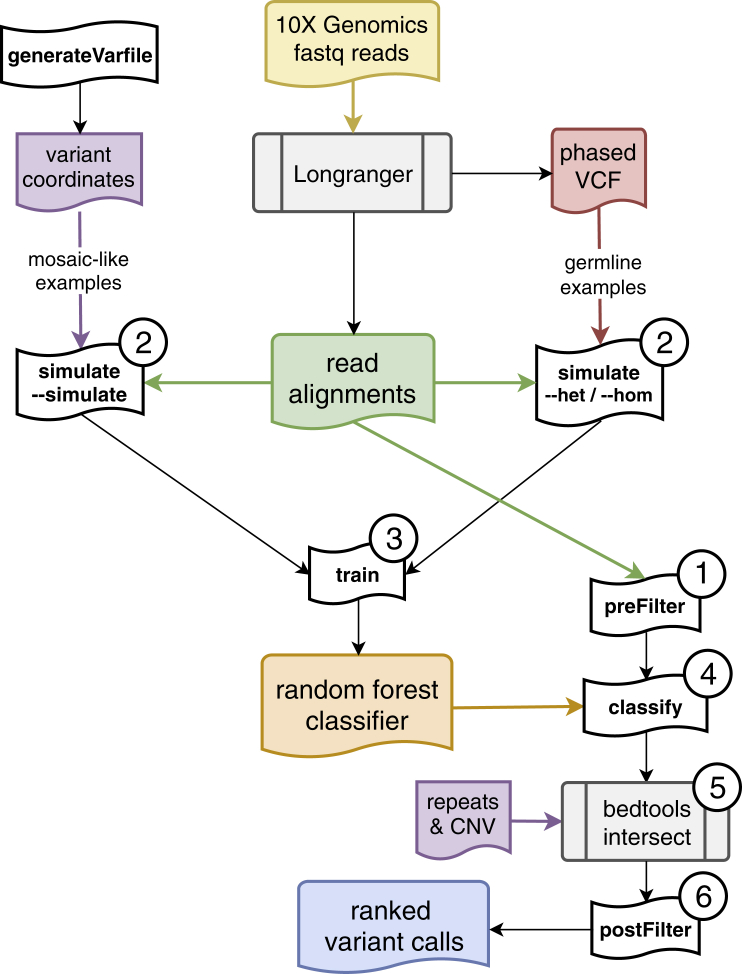
Samovar Workflow

The Samovar workflow is shown in Figure 2 and proceeds in six major steps. In step 1, Samovar identifies all genomic sites where there are sufficient data to apply our model. This is done by filtering based on features such as depth of coverage, fraction of reads that are phased, frequency of the candidate mosaic allele, and related data characteristics. In step 2, Samovar modifies the input BAM file to introduce synthetic mosaic variants to be used as sample-specific training data. Specifically, these variants are used as positive examples for training our model, whereas real homozygous/heterozygous variants, as called by Long Ranger, are used as negative examples. In step 3, Samovar trains a random forest model containing an ensemble of 100 individual decision trees that scores sites according to their resemblance to the synthetic-mosaic sites. In step 4, Samovar scores all sites that passed the initial filter using this model. In step 5, complex repeat regions and non-diploid copy-number regions are optionally filtered out. In step 6, a final filter removes false positives resulting from alignment errors to produce scored mosaic variant calls.

### Simulated Dataset

To benchmark Samovar, we used bamsurgeon (Ewing et al., 2015) to insert synthetic mosaic variants into the NA24385 10X Genomics Chromium BAM file from the Genome in a Bottle (GIAB) project (Zook et al., 2016). Training and testing occurred using sites on the autosomal chromosomes only since NA24385 is male, and the training used an independent set of synthetic variants from those used for the evaluation. The mean inferred linked-read length is 16,176 bp with standard deviation 54,387 bp. To evaluate performance at lower coverage and in other tools' tumor/normal “paired” mode, the original BAM file (mean coverage 61.8; median 60 at bamsurgeon-modified sites, excluding reads marked duplicate) was split in half based on read group tag and we subsequently modified only one-half with bamsurgeon (mean coverage 30.6, median 29 at bamsurgeon-modified sites). Splitting by read group tag ensures that an entire linked read will be placed into the derivative BAM file. Experiments with the original BAM file are referred to as “60X coverage” and those with the subsample as “30X coverage.”

#### Samovar Model Comparison

To measure the specific advantage conferred by linked reads, we also implemented two reduced Samovar models that incorporate less of the variant phasing information. The “short-only” model redefines the fragment-level model features so that they use information summarized over the shorter, paired-end-level fragments rather than the longer linked-read-level fragments. In this model, a paired-end read is assigned to a haplotype only if one of the ends overlaps a heterozygous variant phased by Long Ranger. Past work showed that even the phasing information from short fragments can improve mosaic variant calling accuracy (Usuyama et al., 2014). We find that, although the precision is comparable with that of the Samovar full model, the number of variant calls is much lower, resulting in a genome-wide recall of 2.0% at 30X and 60X, because there are few sites for which adequate phasing information can be compiled from short reads alone (Figure S4, Table S4).

We also created a “no-phasing” Samovar model that used no fragment phasing information at all. This was accomplished simply by omitting the fragment-level features from the model. When stratified by mosaic allele frequency (MAF), precision in every bin is near zero, although genome-wide recall is 68.3%, underscoring the importance of phasing features to our approach (Figure S4, Table S4).

#### MosaicHunter and MuTect2 Comparison

We compared Samovar with MosaicHunter v. 1.1 (Huang et al., 2017). We ran MosaicHunter in “tumor-only mode” analyzing only the bamsurgeon-mutated BAM file from NA24385, as well as in “trio mode” where the unaltered GIAB 10X Genomics Chromium BAM files from the mother (NA24143) and father (NA24149) were also provided. The parental BAM files were similarly produced by Long Ranger but not modified by bamsurgeon. Although Samovar does not use trio information, we hypothesized that its modeling of linked reads would allow it to have competitive accuracy. The modified and unmodified halves of the BAM file split by read group were provided when MosaicHunter was run in “paired-mode” as tumor and normal, respectively.

We also compared Samovar with MuTect2 from GATK v. 4.0.12.0 (Cibulskis et al., 2013). We ran MuTect2 in “tumor-only mode” and tumor/normal “paired-mode” on the same data described earlier. Tumor-only mode calls mosaic and germline mutations simultaneously but does not differentiate between the categories; hence the number of calls is much higher and the precision suffers at higher MAF where germline heterozygous variants comprise most of the call set.

**Figure 3:**
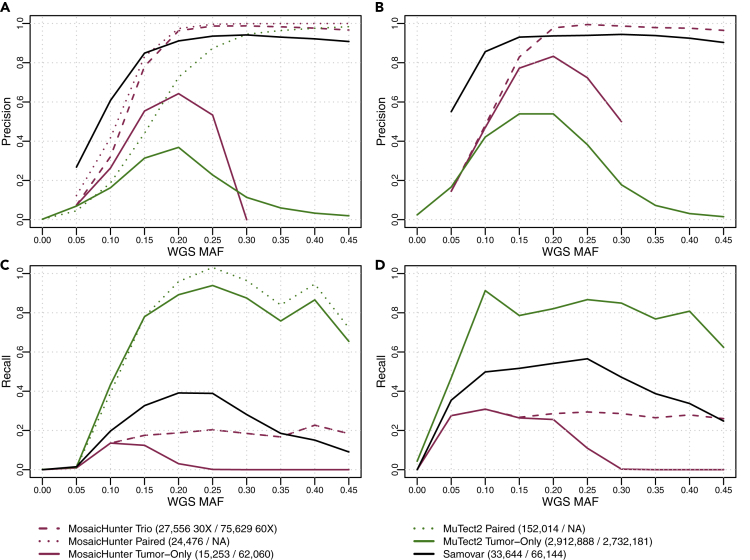
Precision and Recall Calculated for Samovar, MuTect2, and MosaicHunter Variant Calls Stratified by Mosaic Allele Fraction (MAF) in the Whole-Genome Sequencing Data (WGS) (A–D) (A) 30X coverage, precision; (B) 60X coverage, precision; (C) 30X coverage, recall; (D) 60X coverage, recall.

Figure 3 shows each tool's precision and recall, stratified by MAF in the tumor WGS. Precision is calculated as the fraction of variant calls made that were bamsurgeon synthetic mutations, and recall is calculated as the fraction of bamsurgeon synthetic mutations that were in each tool's variant call set. Samovar achieves consistently higher precision than the tumor-only modes of MuTect2 and MosaicHunter. Importantly, Samovar's precision is also comparable with that of those tools in their trio and paired modes, with MosaicHunter's paired and trio modes achieving slightly higher precision at MAFs ≥ 0.2 and MuTect2's paired mode achieving higher precision at MAFs ≥ 0.3.

Note that in all cases, the original 10X Genomics BAM file was used. This means that all three Samovar models (as well as MuTect2 and MosaicHunter) benefited from the improved alignment accuracy of the linked-read-aware Lariat aligner, giving the short-only and no-phasing models and the other two methods a somewhat artificial advantage.

In addition to performance genome-wide we evaluated precision and recall (i.e., TPR) across different annotated genomic regions: genes, exons, all repeats, Alu repeats, segmental duplications, enhancers, and promoters listed in the UCSC Genome Browser and Ensembl, shown in Table 1. Recall is calculated as the fraction of bamsurgeon synthetic mutations with at least four mosaic allele reads that were in the variant call set since both Samovar and MosaicHunter require at least four reads to support a variant call. In practice, many tools including Samovar and MosaicHunter apply filters that exclude portions of the genome that lack sufficient evidence or that are inherently difficult to analyze, such as highly repetitive portions, which particularly contributes to MosaicHunter's poor performance in these genomic regions (see “Genomic regions and filters”). Furthermore, 66% of the Samovar false-negative sites over which recall was evaluated in the 30X coverage experiment and 38% of false negatives in the 60X experiment had fewer than four haplotype-discordant reads, which is the default requirement for Samovar. Relaxing this parameter can boost recall, although it may also impact precision.

**Table 1:** Precision (Prec), Recall (Rec), and F Score of Each Tool for the Synthetic Mosaic Variants Inserted by Bamsurgeon

30X Coverage	Samovar			MuTect2						MosaicHunter								
				Tumor-Only			Paired			Tumor-Only			Paired			Trio		
	Prec	Rec	*F*	Prec	Rec	*F*	Prec	Rec	*F*	Prec	Rec	*F*	Prec	Rec	*F*	Prec	Rec	*F*
Autosomes	84.0	30.1	44.4	3.0	83.2	5.7	60.8	91.4	73.0	31.5	5.1	8.8	79.2	20.7	32.8	70.4	20.7	32.0
Exons	84.0	28.3	42.4	3.6	85.3	7.0	60.1	92.0	72.7	35.0	7.1	11.8	82.1	30.8	44.8	73.7	30.8	43.4
Genes	84.9	30.1	44.4	3.2	84.4	6.2	63.0	92.0	74.8	32.6	5.7	9.7	79.9	22.7	35.4	71.2	22.7	34.5
Enhancer	88.5	31.0	45.9	3.9	86.7	7.5	72.9	92.3	81.4	37.8	5.9	10.1	85.5	29.5	43.8	80.2	29.5	43.1
Promoter	83.3	26.1	39.8	3.0	83.2	5.8	59.4	90.9	71.9	35.3	6.1	10.4	80.5	25.1	38.3	73.7	25.1	37.5
Alu	82.0	28.6	42.4	2.3	78.2	4.5	54.5	88.4	67.4	8.6	0.0	0.1	56.5	0.3	0.6	53.1	0.3	0.6
RepeatMasker	84.2	29.6	43.9	2.8	81.5	5.3	58.9	90.1	71.2	20.2	0.3	0.6	72.3	1.4	2.7	61.3	1.4	2.7
Seg. Dup.	25.6	10.4	14.8	1.3	56.9	2.5	18.4	62.8	28.5	6.6	0.5	0.9	39.3	1.7	3.2	29.1	1.7	3.2

60X coverage	Prec	Rec	*F*	Prec	Rec	*F*				Prec	Rec	*F*				Prec	Rec	*F*

Autosomes	84.6	43.0	57.1	3.6	76.0	7.0				32.4	15.5	20.9				46.8	27.2	34.4
Exons	84.3	41.8	55.9	4.7	79.6	8.8				38.5	25.3	30.5				54.0	45.5	49.4
Genes	85.6	43.4	57.6	3.9	77.2	7.5				33.1	17.0	22.4				47.7	30.0	36.8
Enhancer	90.8	47.8	62.6	4.8	77.9	9.0				36.9	22.7	28.1				51.6	40.0	45.1
Promoter	85.4	40.7	55.2	4.0	76.8	7.6				38.5	21.1	27.3				56.4	40.5	47.2
Alu	81.1	42.9	56.1	3.0	68.0	5.7				16.5	0.2	0.5				31.7	0.5	1.0
RepeatMasker	84.2	42.2	56.2	3.4	74.1	6.4				24.7	1.0	1.9				38.3	1.8	3.4
Seg. Dup.	28.0	13.1	17.8	1.6	48.5	3.1				9.8	1.5	2.6				18.5	2.7	4.7

### Pediatric Cancer Dataset

We next studied a collection of 13 pediatric cancer cases that we sequenced—both tumor and normal—using 10X Genomics Chromium WGS and Whole-Exome Sequencing (WES). One of these cases was studied previously (Miller et al., 2018), and the other twelve are novel to this work. We ran Samovar, MosaicHunter (in both paired and tumor-only modes), and MuTect2 (in both paired and tumor-only modes) on each of the 13 tumor WGS datasets. When running MosaicHunter or MuTect2 in paired mode, we also provided the paired normal WGS.

To estimate accuracy of the different approaches, we used the WES sequencing as a validation dataset as it provides independent and deeper coverage over candidate variants within the exome. We first identified the calls from each tool within the exome capture region. The number and precision of the exome-coincident calls made by each tool are shown in Table 2.

**Table 2:** Number of Variant Calls in the Exome Capture Regions and Precision (Prec) Based on Supporting Reads Found in WES

Case	Samovar		MuTect2				MosaicHunter			
	Full Model		Tumor-Only		Paired		Tumor-Only		Paired	
	Calls	Prec	Calls	Prec	Calls	Prec	Calls	Prec	Calls	Prec
1	22	**0.71**	23,216	0.03	406	0.45	202	0.63	144	0.62
2	23	**0.75**	23,960	0.02	341	0.20	258	0.25	124	0.27
3	42	**0.74**	23,866	0.02	359	0.34	177	0.45	68	0.66
4	37	**0.72**	24,317	0.02	285	0.28	159	0.46	81	0.59
5	21	**0.91**	24,036	0.01	321	0.33	170	0.45	69	0.70
6	50	**0.95**	23,978	0.01	265	0.36	234	0.41	108	0.56
7	23	**0.80**	23,905	0.02	245	0.29	88	0.63	58	0.78
8	28	**0.74**	23,949	0.02	322	0.24	187	0.44	86	0.47
9	25	**0.62**	24,893	0.02	276	0.31	185	0.46	78	0.56
10	29	**0.53**	25,290	0.01	313	0.28	344	0.33	144	0.49
11	22	0.70	24,043	0.02	284	0.41	105	0.75	83	**0.80**
12	21	0.58	23,875	0.02	278	0.48	178	0.58	72	**0.81**
13	15	0.71	23,663	0.02	268	0.35	112	0.76	66	**0.80**
Total	358		312,991		3,963		2,399		1,181	

Samovar has the highest validation rate in 10 of the 13 cases. Bold indicates the highest precision for each pediatric case.

**Figure 4:**
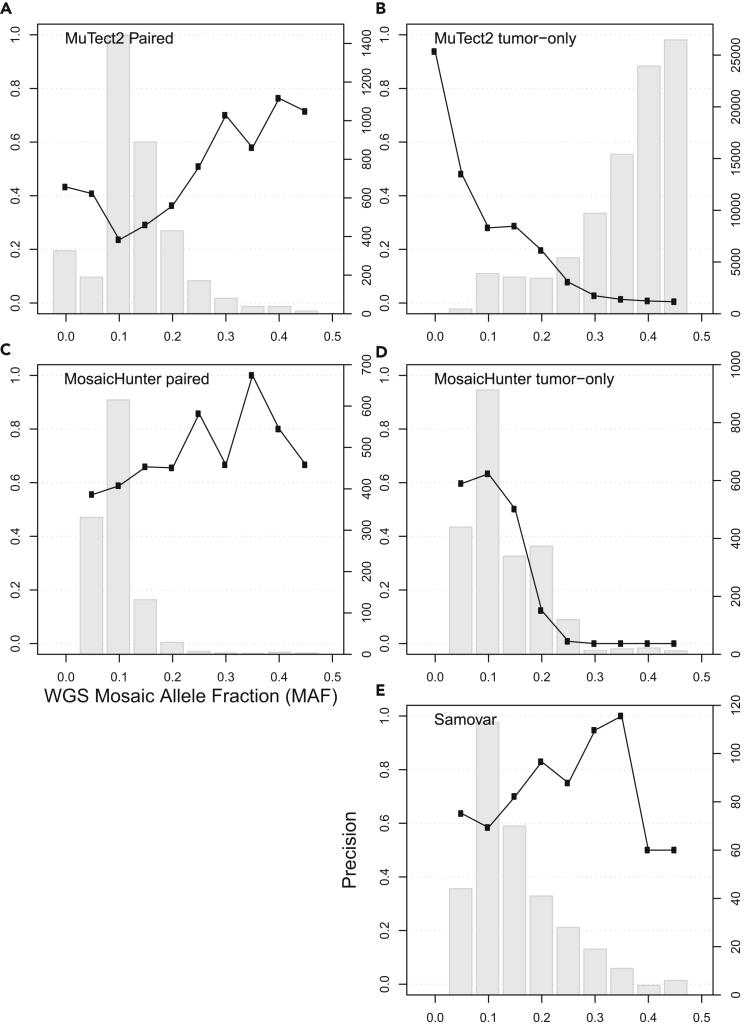
WES Support for Pediatric Cancer Somatic Variant Calls Plots show fraction of variant calls in exome capture region supported by WES data (black line, left axis ticks) and number of variant calls (gray bars, right axis ticks) stratified by mosaic allele fraction (MAF), combined for the 13 pediatric cancer cases studied. The panels show results for (A) MuTect2 Paired, (B) MuTect2 tumor-only, (C) MosaicHunter paired, (D) MosaicHunter tumor-only, and (E) Samovar.

We then examined the corresponding WES tumor data for evidence of the mosaic call made in the WGS data. We considered a mosaic variant call to be “validated” if (1) the corresponding WES tumor sample had at least 50 aligned reads at the locus with at least 4 reads supporting the mosaic allele, and (2) the mosaic variant was not found to be germline by Long Ranger in both the tumor and normal WGS data from that patient. Figure 4 stratifies the validation rate by MAF in the WGS data, and Table 2 shows each tool's overall precision for the calls in the exome capture region. The bar graph shows the number of variants in each MAF bin. MosaicHunter paired called three times as many variants as Samovar, and MuTect2 paired called eleven times as many variants. This is because Samovar requires phasing-based evidence to make a call, which makes it more stringent, and because tumor/normal callers can identify variants that are homozygous or heterozygous in the tumor sample but have a different genotype compared with normal. Additionally, MuTect2 does not filter out CNV regions like MosaicHunter and Samovar, allowing it to call variants in a larger region of the genome. However, Samovar's validation rate is comparable with the paired callers across a range of MAF, indicated by the comparable precision of Samovar in Figure 4E compared with other tools' paired modes in a and c. Against tumor-only modes of other tools, Samovar has superior precision especially at MAF ≥ 0.15: MuTect2 tumor-only mode is not designed to differentiate heterozygous from high-MAF mosaic variants, and MosaicHunter makes few calls with a low validation rate.

As Samovar demonstrated high single-sample precision in simulation, comparable with the other tools' paired analysis, we are also able to run it on the normal control available for each of these cases. Sensitivity was measured in the same fashion using WES of the normal sample; across all 13 samples, 732 variants were in the exome capture region and the validation rate was 65% (see Table S9 for per-sample statistics). More mutations were found in normal samples because a larger fraction of the genome was excluded by CNVNATOR calls in tumor samples, as shown in Table S2. Interestingly, using ANNOVAR (Wang et al., 2010), we determined 11 of these mosaic mutations across 7 cases were nonsynonymous (amino-acid-changing) in one of the 299 cancer driver genes identified in Bailey et al., 2018. The extent of mosaicism in normal tissue and how this may relate to pediatric cancer are interesting avenues of future study now possible with Samovar.

## Discussion

Genomic mosaicism is an important characteristic of many human diseases and conditions. Accurately identifying mosaic variants has previously relied on paired samples or trio analysis, which increases study costs and complexity of studies and may not be possible in many situations. By taking advantage of linked-read properties, particularly the ability to accurately assemble haplotypes, Samovar is able to call mosaic SNVs for a single sample at a level of precision that is comparable with that of paired and trio-based methods. Samovar also achieves substantially higher precision at low MAFs (<15%) and higher recall in more difficult-to-analyze portions of the genome such as segmental duplications and repetitive elements. This opens the door to a wider range of discoveries than are possible with current methods.

Although Samovar already compares favorably to tools that use matched-normal and trio data, in the future it will be important to investigate whether Samovar's recall and precision can be further improved by incorporating trio and matched-normal data directly into its model. Based on the results collected here, we expect that a key benefit of this would be to improve recall at all MAFs and to extend the high precision achieved by the existing paired- and trio-based methods into the low end of the MAF spectrum.

### Limitations of Study

Samovar requires 10X Genomics linked-read data, which currently adds approximately 15% to the cost of a standard paired-end Illumina sequencing experiment. We demonstrate that limited phasing information is available from paired-end reads, but that experiment still used the haplotype phasing information of individual variants from the linked reads and could not be replicated from paired-end reads alone. Finally, although the Samovar model detects only SNPs, it could theoretically be extended to small indels that display the same pattern of haplotype-discordant reads. For this analysis additional indel-related features would also be needed to discriminate true indels from sequencing and alignment errors.

## Methods

All methods can be found in the accompanying Transparent Methods supplemental file.

## Data and Software Availability

The 10X Genomics linked-read whole-genome sequencing (WGS) and whole exome sequencing (WES) data described for the thirteen pediatric cancer cases are available within dbGaP under accession phs001820.v1.p1. The GIAB BAM files with simulated mutations are available at http://share.schatz-lab.org/samovar/simulation.

## Supplemental Information

Document S1. Transparent Methods, Figures S1–S7, and Tables S1–S9

Data S1. Details of Pediatric Cancer Sequencing Data
