# Supplementary material for: Samovar: Single-Sample Mosaic Single-Nucleotide Variant Calling with Linked Reads
Source: iScience. 2019 May 29;18:1–10. doi: 10.1016/j.isci.2019.05.037 (PMC6609817; doi:10.1016/j.isci.2019.05.037)
Supplement: Document S1. Transparent Methods, Figures S1–S7, and Tables S1–S9 [file mmc1.pdf]

ISCI, Volume *18*

Supplemental Information

Samovar: Single-Sample Mosaic Single-Nucleotide Variant Calling with Linked Reads

Charlotte A. Darby, James R. Fitch, Patrick J. Brennan, Benjamin J. Kelly, Natalie Bir, Vincent Magrini, Jeffrey Leonard, Catherine E. Cottrell, Julie M. Gastier-Foster, Richard K. Wilson, Elaine R. Mardis, Peter White, Ben Langmead, and Michael C. Schatz

Transparent Methods

1 Samovar pipeline

Samovar is implemented in Python 3 and operates on the alignment (BAM) and variant (VCF) files produced by 10x Genomics' Long Ranger pipeline. See "Samovar requirements" below for software dependency and input file requirements.

(1) preFilter Samovar first scans the genome calculating the features listed in Figure S8 at each site. Each feature has a numerical threshold, and if all filters are passed the site is considered in step 4 (classify) as a candidate variant site. These filters examine measurements such as depth, number of haplotype-discordant reads, quality of the alignments and credibility of the read phasing.

(2) simulate Simulated mosaic training examples are generated at regular intervals across the genome at a range of mosaic allele frequency (MAF) from 0.025 to 0.475 at increments of 0.025. Such sites are called "simulation sites." Sites harboring germline variant calls can be excluded by specifying them in a VCF. For each phased alignment having the reference allele at the simulation site, the reference allele is randomly changed to the mosaic base with probability equal to the target MAF. For an unphased alignment having the reference allele, the reference allele is randomly changed to the mosaic base with probability $\frac{\text{MAF}}{2}$, on the principle that unphased reads are equally likely to originate from either haplotype. The features listed in Figure S5 are computed for the simulation sites to obtain true-mosaic training examples. The same features are computed for FILTER=PASS phased heterozygous (GT=0|1 or GT=1|0) and homozygous (GT=1|1 or GT= 0|0) variant sites from the VCF to get true-non-mosaic examples.

(3) train A random forest model is trained with an equal number of simulation sites and non-mosaic sites. Non-mosaic sites are selected to have equal amounts of heterozygous and homozygous calls in the VCF. We use the RandomForestClassifier module from the scikit-learn library (Pedregosa et al. 2011) with `max_leaf_nodes 50` and `n_estimators 100`, though Samovar allows the user to customize these hyperparameters. The random forest features described in Table S5 take into account the abundance and consistency of evidence for a mosaic variant, including the number of haplotype discordant reads, mosaic allele fraction, base quality, alignment score, amount of soft clipping, presence of indels, etc.

After cross-validation at a variety of sequencing depths (Table S1), we found that using 20,000 mosaic, 10,000 heterozygous and 10,000 homozygous training examples achieved a balance of computational efficiency and accuracy. We subsampled the NA24385 BAM file used for the simulation experiment and ran the Samovar simulate and train steps. For each number of training examples, average performance statistics are reported for ten independent train/validation splits; 0.5 and 0.9 refer to the random forest probability that the example is in the mosaic class.

(4) classify Genomic sites passing the preFilter are classified by the trained random forest model, yielding the predicted probability that the site is mosaic. Sites with probability above a cutoff are reported in BED format. Based on cross-validation at a variety of sequencing depths, we found that a probability cutoff of 0.5 balances false positive rate and true positive rate (Table S1), though this can be adjusted to trade between sensitivity and precision.

(5) region-based filter As Illumina sequencing is known to have high error rates within microsatellites and simple repeat sequences (Fang et al. 2014), we exclude candidate mosaic variants identified in these regions. Specifically, we exclude variants within +/- 2bp from 1,2,3,4-bp repeats at least 4bp long with at least 3 copies of the unit. Within hg19, 72.0% of autosomes and 71.4% of autosomes+X+Y will remain after this region filter, and within GRCh38 73.8% of autosomes

and 73.1% of autosomes+X+Y remain. We also exclude any CNV regions +/- 5bp identified by CNVNATOR (Abyzov et al. 2011) because polymorphism among the copies of a repeated region would be misconstrued as mosaicism.

(6) postFilter Our expectation is that mosaic variants are isolated events. Samovar applies a final test to distinguish an isolated, likely mosaic variant from the situation where there are many nearby variants co-occurring on the same reads. The latter pattern is usually caused by alignment errors in the presence of repetitive DNA and copy number variation. Specifically, we examine each base within a fixed distance of the mutative mosaic locus. At each base we conduct a Fisher's exact test, testing if the alleles observed at the query base associate with the haplotype-discordant reads. This is diagrammed in Figure S2. If the most significant p-value among all the statistical tests is less than the threshold, the site is filtered out. Based on simulations, we find that the p-value threshold can be set to 0.005 (default) or lower based on the desired balance between precision and recall. There is an option to avoid particular sites when calculating the minimum p-value among all nearby sites and it is recommended to use the germline VCF of variant calls here.

The final mosaic variant calls are reported in VCF format. VCF INFO tags are used to record depth, allele frequency, fraction of reads phased by Long Ranger, number of haplotype-discordant reads, the model-predicted probability, and the minimal p-value obtained by the postFilter.

2 Simulated dataset

Input data We downloaded the 10x Genomics Chromium datasets for the A/J trio processed with Long Ranger version 2.1 and GRCh38 from the GIAB project: FTP Link We use the BAM file from sequencing the son's genome (NA24385) as the basis for this simulation experiment, but MosaicHunter uses the BAM files for the mother (NA24143) and father (NA24149) in trio mode.

We use a custom fork of bamsurgeon (Ewing et al. 2015) to edit the reads in the BAM file. Given a target MAF, a 2 $\times$ MAF fraction of reads with tag HP=1, and a MAF fraction of reads with no HP tag are selected to mutate. The alternate allele is chosen randomly among the three non-reference bases.

Simulated mosaic mutations were introduced at evenly spaced intervals every 20,000 bp on the autosomes with target MAF between 0.025 and 0.475 in increments of 0.025. Reads were realigned with BWA-MEM after mutations were introduced. To compute precision, the denominator is sites with at least 4 alt-allele reads and 16 total reads (not marked duplicate or QC fail). This is because the parameters we chose for Samovar and MosaicHunter require at least 4 reads to call a mosaic variant, and Samovar's depth filter threshold is 16 (MosaicHunter's minimum depth is 25, which we keep, so technically fewer sites are visible to MosaicHunter).

Samovar We use 20,000 simulated mosaic, 10,000 heterozygous and 10,000 homozygous training examples to train each random forest model described. Table S3 has the feature importances of the Samovar model, with abbreviation and number as in Figure S5.

Samovar Short-read phasing model Samovar is designed to take advantage of the long-range phasing information given by linked reads. Previous methods similarly took advantage of the shorter-range phasing information given by paired-end sequencing. We can simulate the paired-end strategy in Samovar, allowing us to compare to the linked-read strategy while holding the rest of the pipeline constant. We begin by creating a "short-read phasing" Samovar model that breaks down the linked reads into their constituent paired-end reads and considers only these shorter fragments when compiling linked-read-related features such as haplotype-discordant reads.

Supposing that we have the complete haplotype phasing from Long Ranger, we assign a haplotype to a pair of reads if either mate overlaps at least SNP with a phased genotype in the VCF. Out of 1.91 billion reads, 9.76% of reads could be phased. Only 0.006% of reads overlapped variants but had alleles for conflicting haplotypes – these were not phased. Table S6 has the feature importances of this limited model, with abbreviation and number as in Figure S6.

Samovar No-phasing model While we do not advocate this approach, for the purposes of comparison, we remove all phasing-related features from Samovar to create a "no-phasing" model. Table S7 has the feature importances of this limited no-phasing model, with abbreviation and number as in Figure S7. Filters use the default parameters described in the preFilter feature list (Figure S8).

MosaicHunter Version 1.1. We used the default recommended parameters when possible, except we did not use the misaligned_reads_filter because it was extremely slow. In addition, because we have simulated far more mosaic sites than would be expected in a normal genome, we do not want to penalize MosaicHunter because it deliberately filters mosaic sites that are close to each other so we changed the following parameters:

- `clustered_filter.inner_distance=2000` [default 20000]
- `clustered_filter.outer_distance=2000` [default 20000]

We also adjusted MosaicHunter's supporting read threshold since Samovar requires at least 4 minor (mosaic) allele reads using: `base_number_filter.min_minor_allele_number=4` [default 3]

We used liftOver to transfer the provided `WGS.error_prone.b37.bed` and `all_repeats.b37.bed` to GRCh38 coordinates, and downloaded `dbsnp_human_9606_b150_GRCh38p7` bed files for the `common_site_filter`, `repetitive_region_filter`, `mosaic_filter.dbsnp_file` respectively. CNVNATOR was used to predict regions of copy number variation and this BED file was provided as the `indel_region_filter.bed_file` parameter.

Note that the `homopolymers_filter`, `common_site` and `repetitive_region` BED files leave visible only 32.2% of bases in the GRCh38 autosomes (34.4% including X and Y) to call mosaic variants. For comparison, Samovar considers about 73% of GRCh38 visible.

MuTect2 Version 4.0.12.0. We executed the standard GATK workflow of the Mutect2 program followed by FilterMutectCalls.

Genomic feature analysis knownGene, knownGene exons, RepeatMasker, RepeatMasker Alu, Segmental duplications are from UCSC Table Browser (GRCh38, accessed 10/02/18). Ensembl Enhancer, Ensembl Promoter + flanking are from Release 94. Ensembl FTP Site

3 Pediatric cancer dataset

Genomic DNA samples Peripheral blood and paired tumor samples were obtained from patients enrolled onto the "Nationwide Children's Neuro-Oncology Tumor and Epilepsy Tissue Bank" protocol (IRB16-00777) at Nationwide Children's Hospital. 13 cases with paired blood and tumor derived DNA were extracted following the manufacturers recommendation using the AllPrep Kit for tumors (Qiagen) and Gentra Purgene or QIAamp Kit (Qiagen) for blood samples. Genomic DNA was quantified with the Qubit dsDNA HS Assay Kit (Life Technologies) and diluted to approximately 1 ng/μL final concentration. DNA source and input mass into sample preparation is described in Supplementary File 1.

Sample preparation and sequencing Linked-read whole genome sequencing (WGS) and whole exome sequencing (WES) libraries were generated (Weisenfeld et al. 2017). Partitioning and barcoding high molecular weight (HMW) DNA was performed using a Chromium Controller Instrument (10x Genomics, CA), and Illumina sequencing libraries were prepared following protocols described in the manufacturer's user guide (Chromium Genome Reagent Kits v2 - Rev A). For WES, 250 ng of each 10x linked-reads library was hybridized in pools (see Supplementary File 1) with 3 pmol of the xGEN Exome Research Panel v1.0 (Integrated DNA Technologies, Coralville, IA) per the manufacturers protocol. Post WES enrichment used standard Illumina P5 and P7 primers (Griffith et al. 2015), and PCR cycling is highlighted in Supplementary File 1. Final libraries were quantified by qPCR (KAPA Biosystems Library Quantification Kit for Illumina platforms), diluted to 3 nM and sequenced using a paired-end recipe on the Illumina HiSeq 4000 next-generation sequencing instrument.

Bioinformatic Analysis Cases using reference genome GRCh38 2.1.0 (1, 2, 7, 10, 11) were processed with Long Ranger 2.1.6 and GATK HaplotypeCaller 3.8-0. Samples using reference genome b37 2.1.0 (3, 4, 5, 6, 8, 9, 10, 12) were processed with Long Ranger 2.1.3 and GATK HaplotypeCaller 3.5-0. The sequencing coverage and fraction of the genome identified by the CNVNATOR (Abyzov et al. 2011) calls is recorded in Table S2, and the oncology diagnosis of each case in Table S8.

4 Computational efficiency

We report timing results for the 30X GIAB sample. Samovar completed in 7 hours with 48 parallel threads for the "filter" step and up to 4 parallel threads for other steps. MuTect2 paired mode completed in 136 hours with 48 parallel threads. MosaicHunter tumor-only and trio modes completed in 29 hours each and paired mode completed in 7 hours. Note MosaicHunter does not offer parallelism options. (See "Command line arguments" for details.)

5 Samovar requirements

Samovar is implemented in Python 3 (also compatible with Python 2). It uses several libraries, including pyfaidx, scikit-learn, simplesam, and fisher. As input, Samovar requires the alignment (BAM) and variant (VCF) files produced by 10x Genomics' Long Ranger pipeline. Long Ranger processes the raw Illumina reads and performs linked read-aware alignment with Lariat (Bishara et al. 2015), small variant calling with Freebayes (Garrison and Marth 2012) or GATK (DePristo et al. 2011), structural variant calling, and haplotype assembly. Specifically, Samovar requires that the BAM have the HP (molecule haplotype), AS (Lariat best alignment score), and XS (Lariat second-best alignment score) extra fields, and requires that the VCF have the FILTER column and GT field. Information on the BAM file tags and phased VCF file are available at 10x Genomics.

6 Computational performance

Running time for each tool was listed for the 30X simulation experiment described in the text. Samovar was run on a single machine with 48 cores for the "filter" step and 4 cores for other parallelizable steps, with pypy when possible. Maximum memory usage was 19.2 GB, and the filter step reported 4200% CPU usage when allocated 48 cores. MosaicHunter and MuTect2 were run on a cluster in a scatter-gather format where each chromosome was computed independently and the results were merged. MosaicHunter does not offer parallelism options, although slightly greater

than 100% average CPU usage was seen. On chromosome 1, paired mode used maximum 25.6 GB memory; tumor-only mode used 25.3 GB; trio mode used 25.0 GB. MuTect2 was run with 48 cores for the "native pair HMM," although only 600% CPU usage was seen on average. On chromosome 1, paired mode used maximum 5.7 GB memory; tumor-only mode used 5.5 GB.

7 Command line arguments

MosaicHunter Version 1.1
The tumor-only, paired, and trio configuration file templates provided with the software distribution were used, containing default parameters.

```
java -jar mosaichunter.jar -C [configuration file] -P output_dir=[output directory]
```

MuTect2 - Paired Mode Version 4.0.12.0

```
gatk Mutect2 -R [reference genome] -I [tumor BAM file] -tumor [tumor sample name] -I
[normal BAM file] -normal [normal sample name] -O [MuTect2 VCF file]
--native-pair-hmm-threads 48
gatk FilterMutectCalls -V [MuTect2 VCF file] -O [Filtered VCF file]
grep -v "multiallelic" [Filtered VCF file] | grep -v "0/1/2" | vcftools --vcf -
--remove-indels --remove-filtered-all --recode --recode-INFO-all --out [Final MuTect2
VCF file]
```

MuTect2 - Tumor-only mode Version 4.0.12.0

```
gatk Mutect2 -R [reference genome] -I [tumor BAM file] -tumor [tumor sample name] -O
[MuTect2 VCF file] --native-pair-hmm-threads 48
gatk FilterMutectCalls -V [MuTect2 VCF file] -O [Filtered VCF file]
grep -v "multiallelic" [Filtered VCF file] | grep -v "0/1/2" | vcftools --vcf
- --remove-indels --remove-filtered-all --recode --recode-INFO-all --out [Final MuTect2
VCF file]
```

Samovar

```
samovar generateVarfile --out out.varfile --vcf [Sample VCF] --fai [Reference genome
FAI]
samovar simulate --bam [Sample BAM] --varfile [Sample VCF] --het --max 15000 --nproc
4 > het.features.tsv
samovar simulate --bam [Sample BAM] --varfile [Sample VCF] --hom --max 15000 --nproc
4 > hom.features.tsv
samovar simulate --bam [Sample BAM] --varfile out.varfile --simulate --nproc 4
> mosaic.features.tsv
samovar train --mosaic mosaic.features.tsv --het het.features.tsv --hom hom.features.tsv
samovar preFilter --bam [Sample BAM --nproc 48 > vectors.txt 2> intervalsComplete.txt
samovar classify --clf clf.pkl --vectors vectors.txt > predictions.tsv
bedtools intersect -v -a predictions.tsv -b [Samovar repeat BED file] | bedtools
intersect -v -a stdin -b [CNVNATOR BED file] > regionfiltered.tsv
samovar postFilter --bam [Sample BAM] --bed regionfiltered.tsv --ref [Reference genome]
--vcfavoid [Sample VCF] --nproc 4 --p 0.005 > samovar.vcf
```

8 Genomic regions and filters

In Table 1 and Figure 3 Samovar and MosaicHunter use their respective default filters but we have treated the tools as though they are interrogating roughly the same portion of the genome. Table S5 and Figure S3 attempt to normalize the differences by reporting just those sites that pass both tools' filters. In GRCh38, this is 32.8% of the autosomal sequence, containing MosaicHunter's simple sequence repeat filter and repetitive region bed files, and Samovar's simple sequence repeat filter, as well as any CNV regions identified by CNVNATOR.

References

Pedregosa, F., G. Varoquaux, A. Gramfort, V. Michel, B. Thirion, O. Grisel, M. Blondel, P. Prettenhofer, R. Weiss, V. Dubourg, et al. (2011). "Scikit-learn: Machine Learning in Python". In: *Journal of Machine Learning Research* 12, pp. 2825–2830.

Fang, H., Y. Wu, G. Narzisi, J. A. O'Rawe, L. T. Barron, J. Rosenbaum, M. Ronemus, I. Iossifov, M. C. Schatz, and G. J. Lyon (2014). "Reducing INDEL calling errors in whole genome and exome sequencing data". In: *Genome Med* 6.10, p. 89.

Abyzov, Alexej, Alexander E Urban, Michael Snyder, and Mark Gerstein (2011). "CNVnator: an approach to discover, genotype, and characterize typical and atypical CNVs from family and population genome sequencing." In: *Genome Research* 21.6, pp. 974–84. DOI: 10.1101/gr.114876.110.

Ewing, Adam D, Kathleen E Houlahan, Yin Hu, Kyle Ellrott, Cristian Caloian, Takafumi N Yamaguchi, J Christopher Bare, Christine P'ng, Daryl Waggott, Veronica Y Sabelnykova, et al. (2015). "Combining tumor genome simulation with crowdsourcing to benchmark somatic single-nucleotide-variant detection". In: *Nature Methods* 12.7, pp. 623–630. DOI: 10.1038/nmeth.3407.

Weisenfeld, Neil I, Vijay Kumar, Preyas Shah, Deanna M Church, and David B Jaffe (2017). "Direct determination of diploid genome sequences." In: *Genome Research* 27.5, pp. 757–767. DOI: 10.1101/gr.214874.116.

Griffith, Malachi, Christopher A. Miller, Obi L. Griffith, Kilannin Krysiak, Zachary L. Skidmore, Avinash Ramu, Jason R. Walker, Ha X. Dang, Lee Trani, David E. Larson, et al. (2015). "Optimizing Cancer Genome Sequencing and Analysis". In: *Cell Systems* 1.3, pp. 210–223. DOI: 10.1016/j.cels.2015.08.015.

Bishara, Alex, Yuling Liu, Ziming Weng, Dorna Kashef-Haghighi, Daniel E Newburger, Robert West, Arend Sidow, and Serafim Batzoglou (2015). "Read clouds uncover variation in complex regions of the human genome." In: *Genome Research* 25.10, pp. 1570–80. DOI: 10.1101/gr.191189.115.

Garrison, Erik and Gabor Marth (2012). *Haplotype-based variant detection from short-read sequencing*. eprint: arXiv:1207.3907.

DePristo, M. A., E. Banks, R. Poplin, K. V. Garimella, J. R. Maguire, C. Hartl, A. A. Philippakis, G. del Angel, M. A. Rivas, M. Hanna, et al. (2011). "A framework for variation discovery and genotyping using next-generation DNA sequencing data". In: *Nat. Genet.* 43.5, pp. 491–498.

Supplementary Figures and Tables

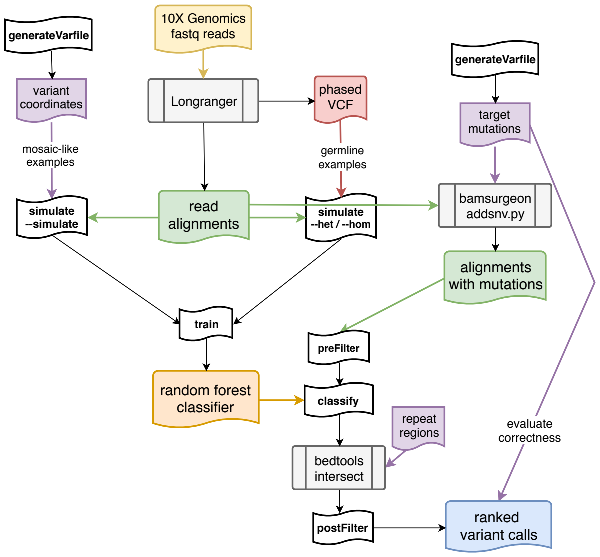

Figure S1: Simulation experiment workflow (left) additionally evaluates correctness of the calls based on mutations generated with bamsurgeon. Related to Figure 2.

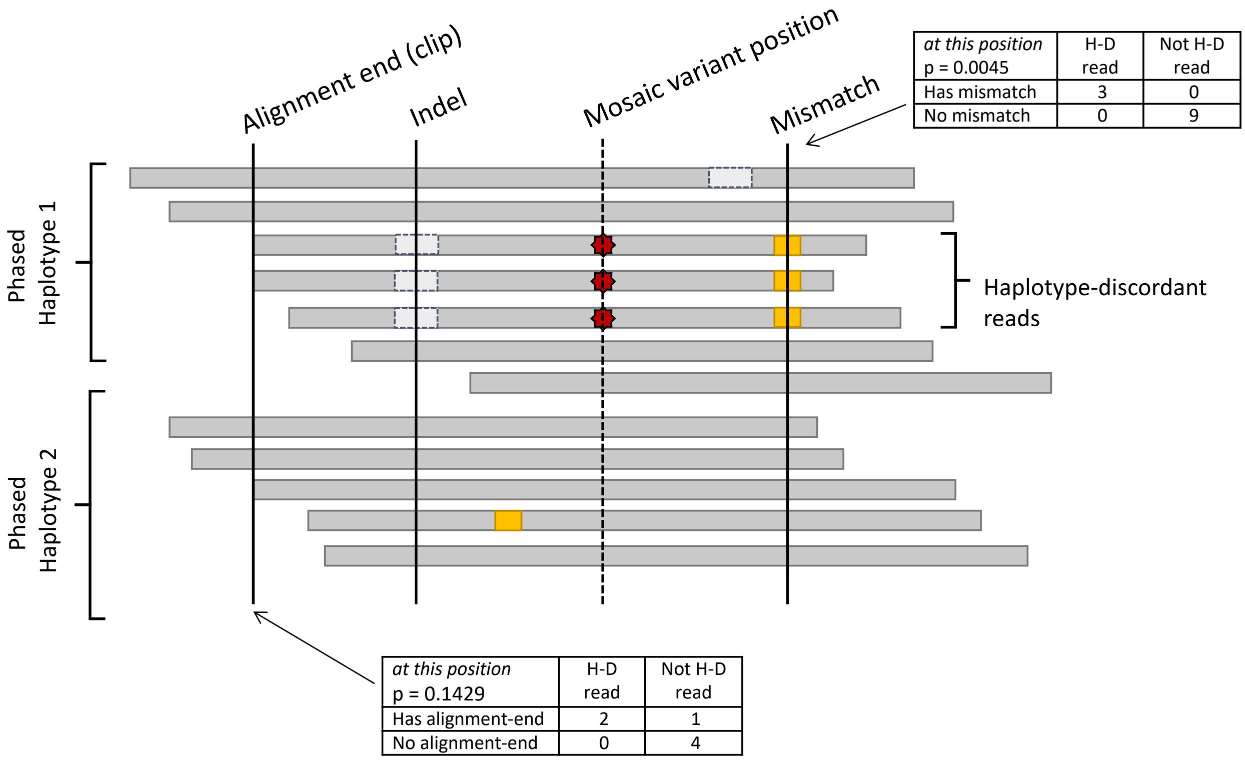

Figure S2: The postFilter step calculates statistical association between haplotype-discordant reads and alignment features such as start/end position, indel or mismatch. Related to Figure 2.

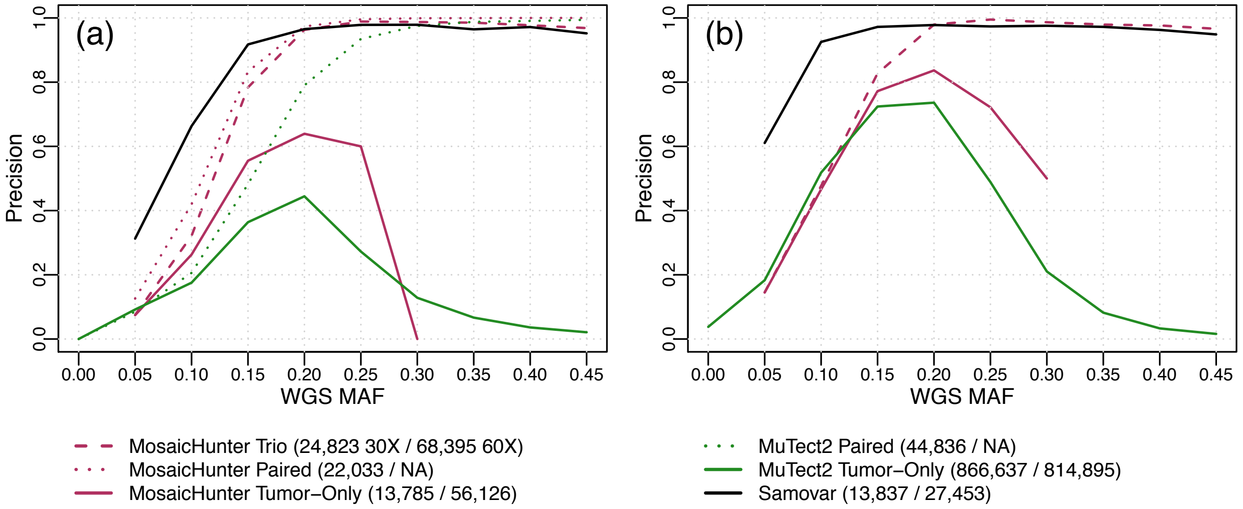

Figure S3: Precision calculated in the genomic region not filtered by MosaicHunter or Samovar's region filters, calculated for Samovar, MuTect2, and MosaicHunter variant calls stratified by mosaic allele fraction (MAF) in whole genome sequencing data (WGS). (a) 30X coverage (b) 60X coverage. Related to Figure 3.

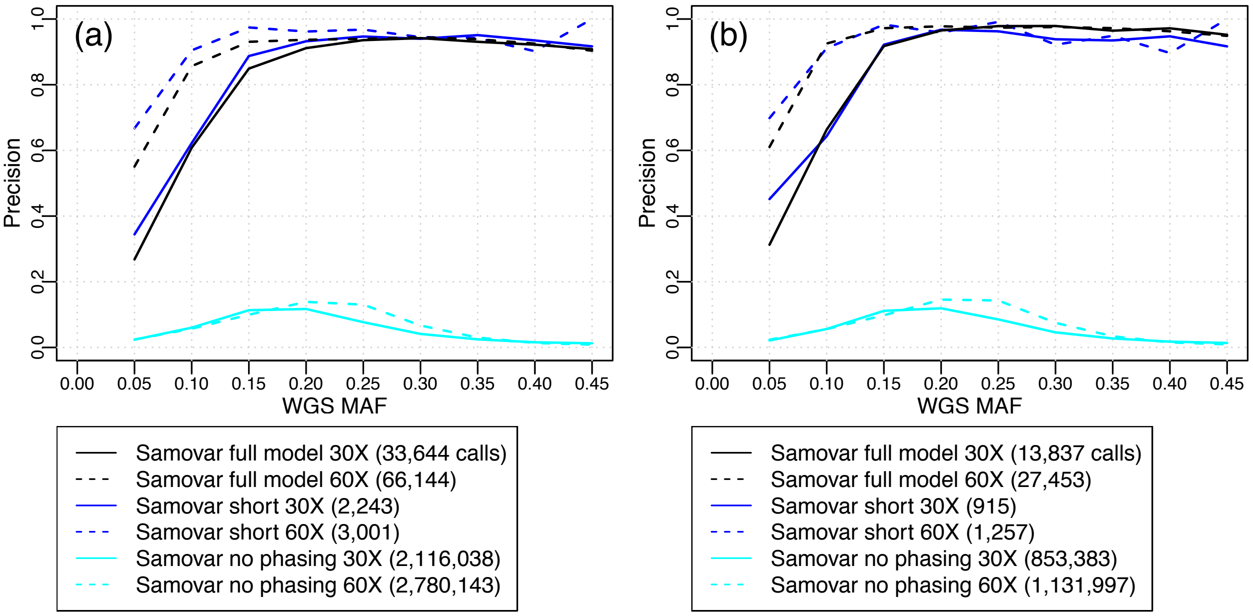

Figure S4: Precision calculated for variant calls made by Samovar's full model and the "short-only" and "no-phasing" models created for illustration, stratified by mosaic allele fraction (MAF) in whole genome sequencing data (WGS). (a) Autosomes (b) Genomic region not filtered by MosaicHunter or Samovar's region filters. Related to Figure 3.

1. Depth [excluding marked duplicates, QC fail, secondary and supplementary alignments]
2. Fraction of reads phased [HP tag assigned by Long Ranger]
3. Fraction of reads on the more common haplotype [max(number of HP=1 reads, number of HP=2 reads)]
4. MAF
5. MAF of phased reads
6. Number of haplotype-discordant (HD) reads
7. Fraction of phased reads that are HD
8. Fraction of HD reads on the more common haplotype [max(number of HP=1 HD reads, number of HP=2 HD reads)]
9. MAF of HD reads
10. Average base quality of HD reads
11. Average position from the closer end of the alignment on HD reads of the site being classified
12. Average number of soft-clipped bases on HD reads
13. Average number of indels in alignment of HD reads
14. Average value of AS – XS (Lariat alignment scores) of HD reads
15-21. Features 8-14 for the set of phased reads that are not HD
22-26. Features 10-14 for the set of mosaic-allele reads
27-31. Features 10-14 for the set of reference-allele reads
32. "weighted" HD read base quality: sum of HD read base quality / sum of all phased reads base quality
33. "weighted" mosaic-allele read base quality: sum of mosaic-allele read base quality / sum of reference- and mosaic-allele read base quality

Figure S5: Samovar random forest features. Related to Figure 2.

1. Depth [excluding marked duplicates, QC fail, secondary and supplementary alignments]
2. Fraction of reads phased [computed based on read or its mate overlapping phased variants]
3. Fraction of reads on the more common haplotype [max(number of HP=1 reads, number of HP=2 reads)]
4. MAF
5. MAF of phased reads
6. Number of haplotype-discordant [HD] reads
7. Fraction of phased reads that are HD
8. Fraction of HD reads on the more common haplotype [max(number of HP=1 HD reads, number of HP=2 HD reads)]
9. MAF of HD reads
10. Average base quality of HD reads
11. Average position from the closer end of the alignment on HD reads of the site being classified
12. Average number of soft-clipped bases on HD reads
13. Average number of indels in alignment of HD reads
14-19. Features 8-13 for the set of phased reads that are not HD
20-23. Features 10-13 for the set of mosaic-allele reads
24-27. Features 10-13 for the set of reference-allele reads
28. "weighted" HD read base quality: sum of HD read base quality / sum of all phased reads base quality
29. "weighted" mosaic-allele read base quality: sum of mosaic-allele read base quality / sum of reference- and mosaic-allele read base quality

Figure S6: Random forest features used in the "short-only" model. Related to Figure 2.

1. Depth [excluding marked duplicates, QC fail, secondary and supplementary alignments]
2. MAF
3. Average base quality of mosaic-allele reads
4. Average position from the closer end of the alignment on mosaic-allele reads of the site being classified
5. Average number of soft-clipped bases on mosaic-allele reads
6. Average number of indels in alignment of mosaic-allele reads
7-10. Features 3-6 for the set of reference-allele reads
11. "weighted" mosaic-allele read base quality: sum of mosaic-allele read base quality / sum of reference- and mosaic-allele read base quality

Figure S7: Random forest features used in the "no-phasing" model. Related to Figure 2.

Median depth of mosaic sites							
**13**	# training examples	**1000**	**2000**	**5000**	**10000**	**20000**	
(mindepth = 14)	Mosaic >0.5	0.9011	0.9013	0.90304	0.90524	0.90627	
	Mosaic >0.9	0.75662	0.7672	0.77045	0.77511	0.77566	
	Het <0.5	0.94845	0.9491	0.94826	0.94843	0.94747	
	Het <0.9	0.98975	0.9895	0.98991	0.99036	0.9898	
	Hom <0.5	0.95197	0.9553	0.95669	0.95762	0.95648	
	Hom <0.9	0.99244	0.9917	0.99265	0.99315	0.99284	
**25**	# training examples	**1000**	**2000**	**5000**	**10000**	**20000**	**30000**
	Mosaic >0.5	0.92024	0.9185	0.92068	0.92388	0.92355	0.92352
	Mosaic >0.9	0.80002	0.8101	0.81813	0.81823	0.81985	0.81635
	Het <0.5	0.9598	0.9608	0.96178	0.96032	0.95997	0.96035
	Het <0.9	0.99254	0.9912	0.99108	0.99168	0.99144	0.99174
	Hom <0.5	0.95935	0.9636	0.96356	0.96434	0.96431	0.96501
	Hom <0.9	0.99462	0.9938	0.99384	0.99436	0.99528	0.99523
**37**	# training examples	**1000**	**2000**	**5000**	**10000**	**20000**	**30000**
	Mosaic >0.5	0.93072	0.933	0.93332	0.93238	0.93283	0.93262
	Mosaic >0.9	0.82435	0.8366	0.84427	0.84729	0.84162	0.84557
	Het <0.5	0.96298	0.9658	0.96576	0.96782	0.96777	0.96736
	Het <0.9	0.99293	0.9924	0.99243	0.99187	0.99252	0.9924
	Hom <0.5	0.96414	0.9685	0.96835	0.97036	0.96997	0.97156
	Hom <0.9	0.99545	0.9951	0.99521	0.99496	0.99551	0.99578
**50**	# training examples	**1000**	**2000**	**5000**	**10000**	**20000**	**30000**
	Mosaic >0.5	0.94165	0.9393	0.94284	0.94151	0.94403	0.94246
	Mosaic >0.9	0.84033	0.858	0.86627	0.86898	0.87017	0.86613
	Het <0.5	0.97008	0.9709	0.97114	0.97159	0.97119	0.97202
	Het <0.9	0.99502	0.994	0.99365	0.99311	0.99345	0.99341
	Hom <0.5	0.96994	0.9733	0.97322	0.97372	0.97408	0.97418
	Hom <0.9	0.99629	0.996	0.99599	0.99566	0.99611	0.99631
**62**	# training examples	**1000**	**2000**	**5000**	**10000**	**20000**	**30000**
	Mosaic >0.5	0.95066	0.9508	0.95103	0.94975	0.9523	0.95019
	Mosaic >0.9	0.86295	0.8777	0.88112	0.88244	0.88564	0.88039
	Het <0.5	0.97127	0.9725	0.97229	0.97518	0.9736	0.97379
	Het <0.9	0.99523	0.9938	0.99411	0.99414	0.99471	0.99444
	Hom <0.5	0.96965	0.9746	0.97586	0.9779	0.97693	0.97807
	Hom <0.9	0.99668	0.9963	0.99674	0.99675	0.99694	0.99707

Table S1: Cross-validation to evaluate the number of training examples and the random forest score threshold. Related to Figure 2.

1. Minimum depth (excluding marked duplicates, QC fail, secondary and supplementary alignments) [at least 16]
2. Minimum fraction of reads phased [at least 0.5]
3. Minimum fraction of reads on less-prevalent haplotype [at least 0.3]
4. Maximum fraction of reads that have neither reference nor mosaic allele [at most 0.05]
5. Minimum mosaic allele frequency [at least 0.05]
6. Minimum number of haplotype-discordant reads [at least 4]
7. Maximum number of haplotype-discordant reads on the less-prevalent haplotype [at most 0.1]
8. Minimum average position from end of alignment of haplotype-discordant reads [at least 10]

Filters that can be “on” or “off”:
1. At least one haplotype-discordant read, one haplotype-concordant read, one reference-allele read and one mosaic-allele read must be aligned in proper pair orientation
2. At least one haplotype-discordant read, one haplotype-concordant read, one reference-allele read and one mosaic-allele read must have an alignment that is not soft-clipped
3. At least one haplotype-discordant read, one haplotype-concordant read, one reference-allele read and one mosaic-allele read must be aligned on the plus and on the minus strand

Figure S8: preFilter features [default value to pass filter in brackets]. Related to Figure 2.

Case	Tumor			Normal		
	WES coverage	WGS coverage	CNVNATOR %	WES coverage	WGS coverage	CNVNATOR %
1	549	45	9.3	617	42	8.7
2	504	41	16.8	529	41	9.3
3*	271	35	23.6	255	34	11.0
4*	223	34	12.4	232	34	11.9
5*	207	34	15.1	268	35	10.9
6*	226	40	11.8	223	38	11.5
7	472	35	10.3	445	38	8.4
8*	330	35	11.1	319	34	10.9
9*	411	36	16.1	346 (Blood)<br>400 (Tissue)	36 (Blood)<br>36 (Tissue)	10.8 (Blood)<br>11.0 (Tissue)
10*	500	40	11.0	392	37	22.0
11	669	37	10.5	579	35	10.5
12*	618	37	11.4	726	37	10.9
13	777	37	20.1	681	37	8.7

Table S2: Cases using reference genome GRCh38 2.1.0 (1, 2, 7, 10, 11) were processed with Long Ranger 2.1.6 and GATK HaplotypeCaller 3.8-0. Samples using reference genome b37 2.1.0 (3, 4, 5, 6, 8, 9, 10, 12) were processed with Long Ranger 2.1.3 and GATK HaplotypeCaller 3.5-0. Related to Table 2.

Importance	Abbreviation	Number in Figure S5
0.206699	weightedMbq	33
0.136303	MAF	4
0.115912	MAF_phased	5
0.101952	weightedCbq	32
0.078008	fracC	7
0.075791	CMAF	9
0.065965	nC	6
0.058420	Mavgbq	22
0.050114	Cavgbq	10
0.028026	NMAF	16
0.016379	Mavgclip	24
0.009496	MavgASXS	26
0.008695	Mavgind	25
0.007776	NavgASXS	21
0.006754	JavgASXS	31
0.006130	CavgASXS	14
0.003744	Cfrach	8
0.003665	Cavgind	13
0.003250	Cavgclip	12
0.002759	Navgbq	17
0.002578	Navgind	20
0.002276	Javgind	30
0.001835	Javgbq	27
0.001569	Javgclip	29
0.001236	fracphased	2
0.001149	depth	1
0.000996	Navgpos	18
0.000904	Mavgpos	23
0.000504	Cavgpos	11
0.000476	Javgpos	28
0.000405	Navgclip	19
0.000148	frach	3
0.000086	Nfrach	15

Table S3: Samovar model feature importances in simulation experiment. Related to Figure 3.

	Samovar									MuTect2						MosaicHunter								
	Full Model			Short			No Phasing			Tumor-Only			Paired			Tumor-Only			Paired			Trio		
30X Coverage	Prec	Rec	*F*	Prec	Rec	*F*	Prec	Rec	*F*	Prec	Rec	*F*	Prec	Rec	*F*	Prec	Rec	*F*	Prec	Rec	*F*	Prec	Rec	*F*
Autosomes	84.0	30.1	44.4	83.7	2.0	3.9	3.4	68.3	6.4	3.0	83.2	5.7	60.8	91.4	73.0	31.5	5.1	8.8	79.2	20.7	32.8	70.4	20.7	32.0
Exons	84.0	28.3	42.4	85.5	1.8	3.5	4.6	70.7	8.6	3.6	85.3	7.0	60.1	92.0	72.7	35.0	7.1	11.8	82.1	30.8	44.8	73.7	30.8	43.4
Genes	84.9	30.1	44.4	84.5	1.8	3.6	3.9	69.2	7.5	3.2	84.4	6.2	63.0	92.0	74.8	32.6	5.7	9.7	79.9	22.7	35.4	71.2	22.7	34.5
Enhancer	88.5	31.0	45.9	90.9	2.1	4.1	4.4	61.8	8.2	3.9	86.7	7.5	72.9	92.3	81.4	37.8	5.9	10.1	85.5	29.5	43.8	80.2	29.5	43.1
Promoter	83.3	26.1	39.8	76.9	1.4	2.7	4.0	65.2	7.5	3.0	83.2	5.8	59.4	90.9	71.9	35.3	6.1	10.4	80.5	25.1	38.3	73.7	25.1	37.5
Alu	82.0	28.6	42.4	81.1	2.3	4.4	2.7	73.1	5.3	2.3	78.2	4.5	54.5	88.4	67.4	8.6	0.0	0.1	56.5	0.3	0.6	53.1	0.3	0.6
RepeatMasker	84.2	29.6	43.9	82.3	2.0	3.9	2.9	67.0	5.5	2.8	81.5	5.3	58.9	90.1	71.2	20.2	0.3	0.6	72.3	1.4	2.7	61.3	1.4	2.7
Seg. Dup.	25.6	10.4	14.8	51.9	0.8	1.5	0.6	25.5	1.2	1.3	56.9	2.5	18.4	62.8	28.5	6.6	0.5	0.9	39.3	1.7	3.2	29.1	1.7	3.2
60X Coverage	Prec	Rec	*F*	Prec	Rec	*F*	Prec	Rec	*F*	Prec	Rec	*F*				Prec	Rec	*F*				Prec	Rec	*F*
Autosomes	84.6	43.0	57.1	87.8	2.0	4.0	3.2	67.9	6.1	3.6	76.0	7.0				32.4	15.5	20.9				46.8	27.2	34.4
Exons	84.3	41.8	55.9	87.3	1.7	3.2	4.6	69.4	8.7	4.7	79.6	8.8				38.5	25.3	30.5				54.0	45.5	49.4
Genes	85.6	43.4	57.6	89.1	2.0	3.8	4.0	68.9	7.5	3.9	77.2	7.5				33.1	17.0	22.4				47.7	30.0	36.8
Enhancer	90.8	47.8	62.6	93.3	2.2	4.2	4.4	61.1	8.1	4.8	77.9	9.0				36.9	22.7	28.1				51.6	40.0	45.1
Promoter	85.4	40.7	55.2	83.1	1.5	2.9	4.0	64.5	7.5	4.0	76.8	7.6				38.5	21.1	27.3				56.4	40.5	47.2
Alu	81.1	42.9	56.1	84.6	2.5	4.8	2.6	72.7	5.0	3.0	68.0	5.7				16.5	0.2	0.5				31.7	0.5	1.0
RepeatMasker	84.2	42.2	56.2	87.1	2.1	4.1	2.6	66.6	5.0	3.4	74.1	6.4				24.7	1.0	1.9				38.3	1.8	3.4
Seg. Dup.	28.0	13.1	17.8	64.3	0.7	1.3	0.5	23.6	1.0	1.6	48.5	3.1				9.8	1.5	2.6				18.5	2.7	4.7

Table S4: Precision (Prec), recall (Rec), and F score of each tool for the synthetic mosaic variants inserted by bamsurgeon. This table includes the Samovar "short" and "no-phasing" models, engineered to demonstrate the importance of linked reads for recall and phasing information for precision. Related to Table 1.

	Samovar									MuTect2						MosaicHunter								
	Full Model			Short			No Phasing			Tumor-Only			Paired			Tumor-Only			Paired			Trio		
30X Coverage	Prec	Rec	*F*	Prec	Rec	*F*	Prec	Rec	*F*	Prec	Rec	*F*	Prec	Rec	*F*	Prec	Rec	*F*	Prec	Rec	*F*	Prec	Rec	*F*
Autosomes	89.6	42.1	57.3	86.9	2.7	5.2	3.6	94.1	7.0	3.2	85.6	6.2	66.1	93.2	77.4	31.6	14.8	20.2	79.3	59.4	67.9	70.5	59.4	64.5
Exons	93.8	39.6	55.7	83.7	2.1	4.2	5.6	94.7	10.6	4.1	87.2	7.9	64.7	93.4	76.4	34.9	12.5	18.4	82.4	54.2	65.3	73.9	54.2	62.5
Genes	90.9	42.4	57.8	87.9	2.5	4.8	4.6	94.8	8.7	3.4	86.7	6.6	67.1	93.7	78.2	32.7	15.1	20.6	80.0	60.1	68.6	71.2	60.2	65.2
Enhancer	94.2	42.0	58.1	91.7	2.5	4.9	5.5	96.1	10.4	4.0	87.7	7.7	70.1	93.6	80.2	36.4	11.3	17.3	85.9	58.7	69.7	80.1	58.7	67.8
Promoter	91.4	36.3	52.0	76.7	1.7	3.4	4.6	93.9	8.8	3.2	85.8	6.2	60.5	92.4	73.1	35.4	11.6	17.5	80.7	48.7	60.7	74.2	48.7	58.8
Alu	30.2	18.6	23.0	25.0	1.2	2.4	0.9	60.5	1.8	1.4	61.4	2.8	27.1	61.4	37.6	9.7	4.3	5.9	52.6	28.6	37.0	50.0	28.6	36.4
RepeatMasker	68.5	33.1	44.7	73.7	2.3	4.4	0.7	73.9	1.3	2.6	72.3	5.0	45.6	75.8	57.0	24.4	10.1	14.2	75.5	45.0	56.4	65.3	45.0	53.3
Seg. Dup.	6.8	4.4	5.3	28.6	0.6	1.2	0.1	17.0	0.2	0.8	42.5	1.6	11.1	39.6	17.4	7.8	4.1	5.4	37.6	11.9	18.1	27.7	12.3	17.0
60X Coverage	Prec	Rec	*F*	Prec	Rec	*F*	Prec	Rec	*F*	Prec	Rec	*F*				Prec	Rec	*F*				Prec	Rec	*F*
Autosomes	89.7	60.3	72.1	89.3	2.7	5.3	3.4	94.0	6.6	4.0	78.7	7.6				32.4	44.5	37.5				46.8	78.3	58.5
Exons	91.7	58.5	71.4	89.2	2.1	4.1	5.9	95.0	11.2	5.4	81.8	10.1				38.6	44.7	41.4				54.0	80.6	64.7
Genes	90.8	60.8	72.8	91.3	2.7	5.2	5.0	95.1	9.4	4.3	79.8	8.1				33.1	45.0	38.1				47.6	79.4	59.5
Enhancer	94.5	63.3	75.8	94.4	2.8	5.5	5.8	94.8	10.9	4.9	79.6	9.3				36.5	45.1	40.4				51.1	79.6	62.3
Promoter	91.2	57.3	70.4	92.2	2.3	4.5	4.9	94.7	9.4	4.2	78.2	8.0				38.6	40.5	39.5				56.5	78.1	65.6
Alu	45.9	30.0	36.3	57.1	3.1	5.8	0.7	53.1	1.4	2.1	56.2	4.1				17.0	20.8	18.7				32.4	43.8	37.3
RepeatMasker	73.6	43.9	55.0	82.7	2.5	4.8	0.4	69.4	0.8	3.2	62.8	6.0				27.5	31.2	29.2				41.5	54.9	47.3
Seg. Dup.	9.2	6.0	7.3	15.4	0.4	0.7	0.1	13.3	0.2	1.0	32.9	2.0				10.1	10.3	10.2				18.8	18.5	18.7

Table S5: Precision (Prec), recall (Rec), and F score of each tool for the synthetic mosaic variants inserted by bamsurgeon in the region of the genome not filtered out by MosaicHunter or Samovar. This table includes the Samovar "short" and "no-phasing" models, engineered to demonstrate the importance of linked reads for recall and phasing information for precision. Related to Table 1.

Importance	Abbreviation	Number in Figure S6
0.20520657	weightedMbq	33
0.16749462	MAF	4
0.13410861	weightedCbq	32
0.08401046	fracC	7
0.07369292	MAF_phased	5
0.06894606	Mavgbq	22
0.06203258	nC	6
0.02828793	Cfrach	8
0.02817725	Mavgclip	24
0.02439597	Cavgbq	10
0.01783998	MavgASXS	26
0.01466872	JavgASXS	31
0.01386411	CMAF	9
0.01311324	CavgASXS	14
0.01274496	NMAF	16
0.00876721	NavgASXS	21
0.008098	Mavgind	25
0.00757443	fracphased	2
0.00331775	Navgind	20
0.00318227	Javgind	30
0.00307518	Cavgind	13
0.00297741	Mavgpos	23
0.00291929	Cavgpos	11
0.00285171	Javgbq	27
0.00209531	Javgclip	29
0.00169362	Navgbq	17
0.00123443	Javgpos	28
0.00070641	Navgclip	19
0.00070569	Nfrach	15
0.00069773	Navgpos	18
0.00069729	depth	1
0.00052806	frach	3
0.00029423	Cavgclip	12

Table S6: Short-read phasing model feature importances in simulation experiment. Related to Figure 3.

**Importance**	**Abbreviation**	**Number in Figure**
0.42298002	weightedMbq	11
0.29348068	MAF	2
0.14507064	Mavgbq	3
0.07905771	Mavgclip	5
0.03207496	Mavgind	6
0.00746506	Javgind	10
0.00448625	Javgpos	8
0.00438944	Javgbq	7
0.00403807	depth	1
0.0038253	Mavgpos	4
0.00313186	Javgclip	9

Table S7: No-phasing model feature importances in simulation experiment. Related to Figure 3.

**Case**	**Diagnosis**	**Sex***
1	Indeterminate, most consistent with oligodendroglioma	M
2	Pilocytic astrocytoma	M
3	Medulloblastoma, WHO grade IV, most consistent with non-WNT, non-SHH subgroup	M
4	Pilocytic astrocytoma	F
5	Glioblastoma (recurrence)	F
6	Pilocytic astrocytoma	F
7	Ewing-like sarcoma	M
8	Ganglioglioma, WHO grade 1	M
9	Diffuse Midline Glioma, H3 K27M-mutant, WHO grade IV	M
10	Indeterminate, high grade glioma/astrocytoma	M
11	Ganglioglioma, WHO grade 1	F
12	Glioma (low grade)	M
13	Clival chordoma	F

Table S8: Metadata for each case. * Sex determined from alignments to Y-chromosome. Related to Table 2.

Case	Calls	Sensitivity
1	58	0.70
2	85	0.61
3	70	0.57
4	73	0.68
5	51	0.58
6	73	0.58
7	61	0.63
8	43	0.62
9	39	0.48
10	50	0.45
11	30	0.73
12	59	0.78
13	70	0.88
Total	762	

Table S9: Samovar analysis of normal WGS dataset for pediatric cancer cases. Number of calls shown is for the WES capture region, and validation performed as described in main text. Related to Figure 4.
